# A PDMS-Based 2-Axis Waterproof Scanner for Photoacoustic Microscopy

**DOI:** 10.3390/s150509815

**Published:** 2015-04-27

**Authors:** Jin Young Kim, Changho Lee, Kyungjin Park, Geunbae Lim, Chulhong Kim

**Affiliations:** 1 Department of Mechanical Engineering, Pohang University of Science and Technology (POSTECH), Pohang 790-784, Korea; E-Mail: ronsan@postech.ac.kr; 2 Department of Creative IT Engineering, Pohang University of Science and Technology (POSTECH), Pohang 790-784, Korea; E-Mail: ch31037@postech.ac.kr; 3 School of Interdisciplinary Bioscience and Bioengineering, Pohang University of Science and Technology (POSTECH), Pohang 790-784, Korea; E-Mail: kjpark@postech.ac.kr

**Keywords:** optical-resolution photoacoustic microscopy, MEMS scanner, polydimethylsiloxane

## Abstract

Optical-resolution photoacoustic microscopy (OR-PAM) is an imaging tool to provide *in vivo* optically sensitive images in biomedical research. To achieve a small size, fast imaging speed, wide scan range, and high signal-to-noise ratios (SNRs) in a water environment, we introduce a polydimethylsiloxane (PDMS)-based 2-axis scanner for a flexible and waterproof structure. The design, theoretical background, fabrication process and performance of the scanner are explained in details. The designed and fabricated scanner has dimensions of 15 × 15 × 15 mm along the X, Y and Z axes, respectively. The characteristics of the scanner are tested under DC and AC conditions. By pairing with electromagnetic forces, the maximum scanning angles in air and water are 18° and 13° along the X and Y axes, respectively. The measured resonance frequencies in air and water are 60 and 45 Hz along the X axis and 45 and 30 Hz along the Y axis, respectively. Finally, OR-PAM with high SNRs is demonstrated using the fabricated scanner, and the PA images of micro-patterned samples and microvasculatures of a mouse ear are successfully obtained with high-resolution and wide-field of view. OR-PAM equipped with the 2-axis PDMS based waterproof scanner has lateral and axial resolutions of 3.6 μm and 26 μm, respectively. This compact OR-PAM system could potentially and widely be used in preclinical and clinical applications.

## 1. Introduction

Photoacoustic microscopy (PAM) has become an important biomedical imaging technique that achieves high-resolution and rich optical contrast by merging optical irradiation and ultrasound detection [1]. When pulsed light illuminates and spreads in biological tissues, targeted biomolecules absorb light and consequently photoacoustic (PA) waves are created via thermoelastic expansion. The produced PA waves are captured by acoustic transducers, and then 1D-, 2D- and/or 3D PA images can be formed to show structural, functional, and molecular information of the tissues [2,3,4,5,6]. Particularly, optical-resolution PAM (OR-PAM) is able to visualize label-free micro-scale images of oxy- and deoxyhemoglobin [7], melanin [8], DNA/RNA in cell nuclei [9], and so on. Due to its strong optical contrast and high optical resolution, OR-PAM has successfully been utilized in many biological studies and applications such as vascular biology [10], neurology [11], ophthalmology [12], intraoperative surgery [13] and so forth. The conventional OR-PAM systems utilize a special opto-ultrasound beam combiner for coaxial and confocal alignment of light illumination and ultrasound detection [14]. This opto-ultrasound beam combiner is the essential part of the conventional OR-PAM systems to reach the maximum signal-to-noise ratios (SNRs). However, the conventional OR-PAM systems suffer from slow imaging speed and are relatively bulky because of linear raster scanning using stepping motors (normally 1 Hz to acquire one depth-resolvable PA B-scan images [15]). However, to expand the usabilities of OR-PAM in preclinical and clinical applications, small size and fast imaging speed are crucial. To improve the imaging speed, microelectromechanical systems (MEMS)-based scanners have been widely developed. Among them silicon-based MEMS scanners have many advantages due to their small sizes and fast scanning speeds. Thus, they have been used in numerous medical imaging applications such as confocal microscopy [16], optical coherence tomography [17], and multi-photon microscopy [18]. Further, the applications have been even more expanded to build handheld probes and endoscopic systems. However, it is difficult to implement the OR-PAM systems with the conventional MEMS scanners because the scanners should be workable in a water environment. In addition, simultaneous co-axial scanning of both light and ultrasound together is another key requirement in order to maintain high SNRs. To address these issues, several approaches have been studied. A 1-axis water immersible scanning mirror has been developed and applied to OR-PAM [19], but it requires an additional bulky linear stage for 2D scanning. Although a 2-axis water-immersible scanning mirror has been introduced [20], the fabrication process includes complex laser cutting of polymer film and manually aligning between two scanning axes. Furthermore, no PA imaging capability has been proved in the report. Recently, our group reports the fast OR-PAM system with a 2-axis water-proofing MEMS scanner [21]. The flowing carbon particles in tube and microvasculatures of a mouse ear are successfully monitored. 

In this paper, we introduce design, theoretical background, fabrication and evaluation processes of a 2-axis polydimethylsiloxane (PDMS)-based waterproof scanner (2A-PDMS-WP-scanner) in the previously reported OR-PAM system in details. The soft and water-resistant properties of PDMS make the scanner useable underwater. Moreover, the fabrication process uses micro-milling and soft lithography, which are relatively easy and inexpensive compared to conventional MEMS processes. We test the scanning characteristics of 2A-PDMS-WP-scanner and adopt it in two types of OR-PAM for evaluation of the sensitivity of system. Finally, we have successfully characterized the 2A-PDMS-WP-scanner by photoacoustically imaging gold micro-patterns *in vitro* and microvasculatures of a mouse ear *in vivo*. 

## 2. Fabrication and Characterization of a 2A-PDMS-WP Scanner

### 2.1. Design of a 2A-PDMS-WP-Scanner

**Figure 1:**
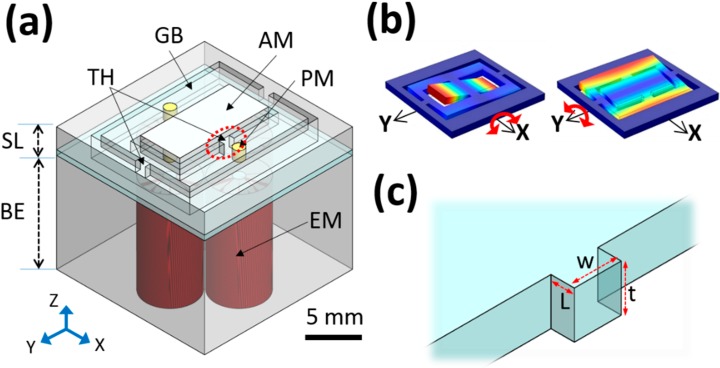
Design of the 2A-PDMS-WP-scanner. (**a**) 3D scheme of the 2A-PDMS-WP-scanner; (**b**) Scheme of torsional motions along the X and Y axes; (**c**) Magnified view of a torsional hinge. SL, structure layer; BE, block of four electromagnets; AM, aluminum mirror; PM, permanent magnets; EM, electromagnets; GB, gimbal; and TH, torsional hinges.

The 2A-PDMS-WP-scanner mainly consists of a movable structure layer of PDMS and a fixed block of four electromagnets (Figure 1a). The movable structure layer has two scanning axes on a single layer along the X and Y axes (Figure 1b). An aluminum (Al)-coated mirror on the center of the structure layer sufficiently reflects both laser and ultrasound with a reflection rate of 92% and 84%, respectively, in water, and can torsionally be moved along the X and Y axes. As we mentioned before, the key requirement for designing the scanner which can be controllable in water is to overcome the strong damping of torsional oscillation by water and prevent electrical leakage from water. Because of its strong mechanical stability and chemical inertness, PDMS is selected as a material for the structure layer. In addition, PDMS itself is well suited for mechanical sensors or actuators such as accelerometers [22]. 

The resonant frequency $f_{t}$, closely related to the scanning speed along the X axis, is calculated as follows:

(1) $$f_{t}=\frac{1}{2\pi }\sqrt{\frac{K_{t}}{J}}$$

where J (kg·m^2^) is the torsional moment of inertia of the mirror, which is calculated as 1 × 10^−9^ kg·m^2^ by mass and size, and $K_{t}$ (N·m) is the torsional stiffness of the hinge:

(2) $$K_{t}=\frac{G}{L}\left[tw^{3}\left\{\frac{1}{3}-0.21\frac{w}{t}\left(1-\frac{w^{4}}{4t^{4}}\right)\right\}\right]$$

where G (Pa) is the shear modulus, L (m) is the length, t (m) is the thickness, and w (m) is the width of the torsional hinge (Figure 1c). Because PDMS has a very low G value of ~250 kPa [23], the designed torsional hinge could have a mechanically strong structure with a length L of 0.5 mm, a thickness t of 1 mm, and a width w of 1 mm. Consequently, the calculated torsional stiffness and resonant frequency are 141 µN·m and 59.2 Hz, respectively. If the supporting material has a high G value like the single crystal silicon used in conventional MEMS fabrication (*i.e.*, ~80 GPa), the torsional hinge needs to be very thin to provide a comparable resonant frequency (*i.e.*, a cross section of 40 µm × 40 µm in width and thickness, respectively). Then, the structure becomes delicate and susceptible to damping, and thus it may be fragile when the scanner vibrates in water. In our design, a rigid gimbal acrylic frame and a mirror plate are assembled to the PDMS body to reinforce the elastic PDMS structure except the torsional hinge. The gimbal structure of the single movable layer reduces mechanical coupling between two torsional movements, and thus helps to enhance the scanning preciseness and linearity [24]. 

**Figure 2:**
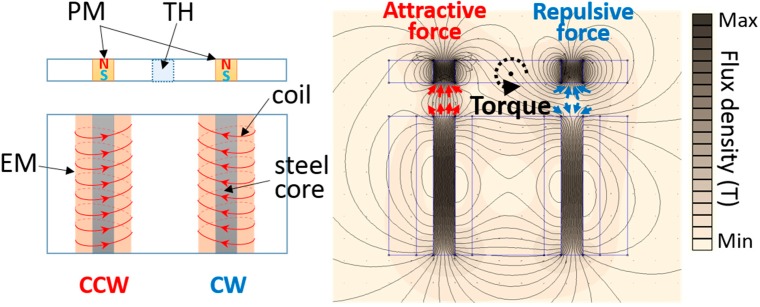
Simulated magnetic field distribution induced by electromagnets within the 2A-PDMS-WP-scanner. PM, permanent magnets; EM, electromagnets; TH, torsional hinges; CCW, counter clock wise; and CW, clock wise.

Two torsional motions are induced by two independent pairs of electromagnetic forces between four permanent magnets and beneath four electromagnets. The induced magnetic field distribution is simulated using the finite element method magnetics (FEMM) as shown in Figure 2 [25]. When electrical current flows in two oppositely winded enameled coils, the induced magnetic field enables two steel cores be electromagnets. These electromagnet pairs generate attractive and repulsive forces with permanent magnets alternately. Then, the consequently stimulated torque at the torsional hinge induces scanning motion along both X and Y axes. 

### 2.2. Fabrication Procedures of the 2A-PDMS-WP-Scanner 

**Figure 3:**
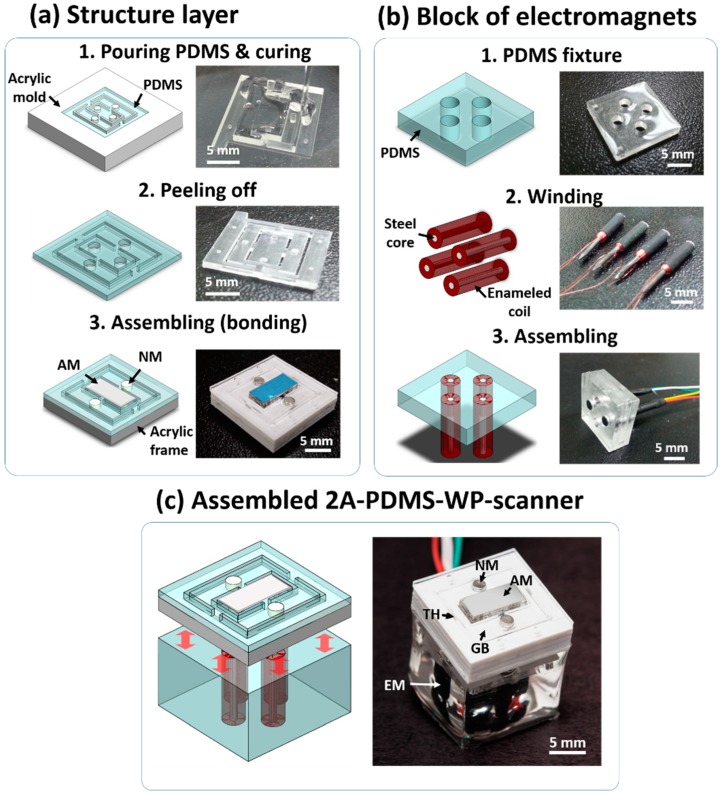
Fabrication process of the 2A-PDMS-WP-scanner. (**a**) Structure layer; (**b**) Block of electromagnet; (**c**) The 2A-PDMS-WP-scanner. NM, neodymium magnets; AM, aluminum mirror; TH, torsional hinges; GB, gimbal; and EM, electromagnets.

The movable structure layer of the 2A-PDMS-WP-scanner is fabricated using a PDMS molding process (Figure 3a). PDMS-based soft lithography is another well-developed MEMS process which is widely used in fabrication of microstructures [26]. Using a micro-milling machine, a mold is constructed from an acrylic plate. The end mill with a size of 200 μm is used to cut the smallest body layer with a dimension of 500 μm. The thin acrylic gimbal frames to support and reinforce the flexible PDMS body are also prepared using this milling process. Then, a mixture of PDMS prepolymer and curing agent (Sylgard^®^ 184, Dow Corning, Midland, MI, USA) is prepared with a weight ratio of 10:1, respectively. The mixture is filled into the acrylic mold and cured in a convection oven at 70 °C for 3 h; the resulting solid PDMS body is carefully peeled off from the mold. E-beam evaporation and dicing are used to fabricate the mirror plate by depositing 200-nm-thick Al on the glass plate. 

Finally, the prepared PDMS body, acrylic gimbal frames, four neodymium magnets, and the mirror plate are assembled together using plasma bonding between PDMS and the supporting structures. The same PDMS process is applied to prepare the PDMS fixture to position electromagnets (Figure 3b). A homemade winding system is used to fabricate four electromagnets by winding enameled copper wires with a thickness of 50 μm. These electromagnets are assembled with the PDMS fixture. An additional PDMS molding process is used to engineer a waterproof capsule for the block of electromagnets. Finally, the fabricated structure layer and block of electromagnets are assembled to the 2A-PDMS-WP-scanner using plasma bonding (Figure 3c). The size of the fabricated 2A-PDMS-WP-scanner is 15 × 15 × 15 mm along X, Y and Z axes, respectively.

### 2.3. Scanning Characteristics of the 2A-PDMS-WP-Scanner 

Two important scanning characteristics of the 2A-PDMS-WP-scanner include the maximum scanning angle and scanning speed; these characteristics are related to the field of view (FOV) and imaging speed in OR-PAM, respectively. These characteristics are determined by the applied amplitude and frequency of driving signals. The driving signals are generated by a data acquisition (DAQ) system (NI PCIe-6321, National Instruments, Austin, TX, USA) and amplified by a high-current operational amplifier (OPA2544, Texas Instruments, Dallas, TX, USA). The scanning angle can be calculated by measuring the scanning length from the center of the mirror plate to the preset positions.

**Figure 4:**
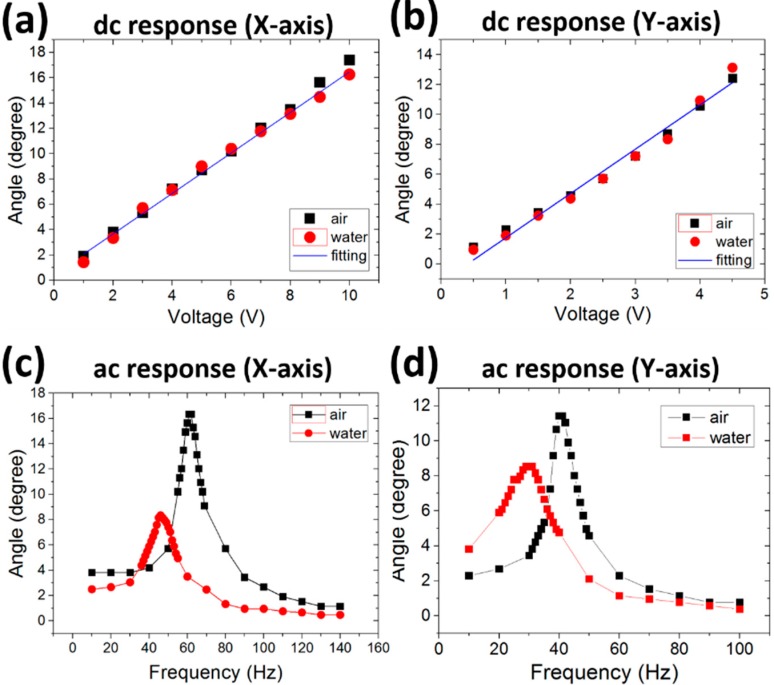
Scanning responses of the 2A-WP-MEMS-scanner in DC and ac conditions. Scanning angles *versus* the applied DC voltages along the (**a**) X and Y (**b**) axes, respectively. Scanning angles *versus* the applied ac frequencies at 2 V along (**c**) X and (**d**) Y axes, respectively.

When DC voltages are applied, the scanning angles increase linearly with the applied voltages in both X and Y axes as shown in Figure 4a,b. Further, the scanning angles in both axes are almost identical in both water and air environments. The maximum scanning angles are 18° at 10 V (Figure 4a) and 13° at 5 V along the X and Y axes (Figure 4b). The corresponding FOVs are 14.2 mm × 10.2 mm along the X and Y axes, respectively, at 22 mm away from the mirror surface where the typical light and ultrasound foci are. Our results imply that the fabricated 2A-PDMS-WP-scanner provides the wide FOV even with DC voltage application due to its low torsional stiffness of the hinge. Although the scanner can work well under the DC condition, the application of an AC voltage gives a fast and wide scanning capability when the scanning speed is near the resonant frequency (Figure 4c,d). In air, the resonance frequencies are 60 and 45 Hz along the X and Y axes, respectively. In water, the resonance frequencies are 45 and 30 Hz along the X and Y axes, respectively, due to damping by water. The damping effect reduces the scanning angle (*i.e.*, 8.5° and 8.5° at 2V along X and Y axes, respectively) in water compared to that in air. The corresponding FOVs are 6.6 mm × 6.6 mm along the X and Y axes, respectively, at 22 mm away from the mirror surface where our targets are.

## 3. *In Vitro* and *in Vivo* Photoacoustic Imaging Using the 2A-WP-MEMS-Scanner

### 3.1. Comparison of Signal-to-Noise Ratios (SNRs)

**Figure 5:**
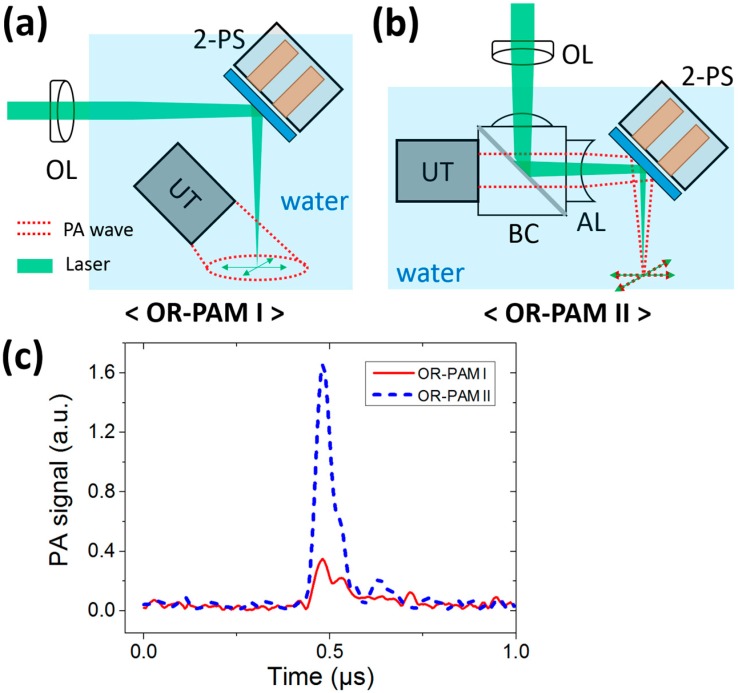
Comparison of signal-to-noise ratios in two forms of OR-PAM. (**a**) Schematic of the OR-PAM I system. An unfocused ultrasound transducer is used and the laser beam is only scanned by the 2A-PDMS-WP-scanner; (**b**) Schematic of the OR-PAM II system. Both light and ultrasound are confocally scanned by the 2A-PDMS-WP-scanner; (**c**) Hilbert-transformed PA A-line profiles of a black vinyl tape acquired by the OR-PAM I and OR-PAM II systems, respectively. UT, ultrasonic transducer; OL, objective lens; BC, beam combiner; AL, acoustic lens; and 2-PS, 2A-PDMS-WP-scanner.

Two forms of the OR-PAM systems are implemented using the 2A-PDMS-WP-scanner (Figure 5). A Q-switched-diode-pumped-solid-state laser (SPOT-10-200-532, Elforlight, Daventry, UK) is commonly used to deliver a 532-nm laser beam (repetition rate, 10 kHz) in all experiments. The first form (OR-PAM I) is developed by only scanning the focused laser beam via an objective lens (AC254-060-A, Thorlabs, Newton, NJ, USA) and the 2A-PDMS-WP-scanner (Figure 5a) [27]. In this mode, an unfocused transducer (10 MHz center frequency, V312, Olympus NDT, Waltham, MA, USA) is utilized to detect the PA waves. The second form (OR-PAM II) requires an additional opto-ultrasound beam combiner to achieve simultaneous confocal scanning of laser and ultrasound (Figure 5b). The focused laser beam is first reflected by an Al film between two right angled prisms in the beam combiner, and then directed to the surface of a sample via the 2A-PDMS-WP-scanner. The induced PA waves are first reflected by the scanner, passed through the opto-ultrasound beam combiner, and detected by an ultrasound transducer (50 MHz center frequency, V214-BB-RM, Olympus NDT). Interestingly, the Al coating of the scanner surface enables to reflect both light and ultrasound, while the Al film between two prisms enables to reflect only light and is transparent to ultrasound. Further, the attached acoustic lens (NT45-384, Edmund, Barrington, NJ, USA) in front of the opto-ultrasound beam combiner can manipulate focused ultrasound. The imaging speeds of both types are identical because they ultimately rely on the laser repetition rate and the scanning speed. However, the SNRs of the second form is much better than that of the first one because the coaxial geometry of light and ultrasound boost the SNRs. By imaging a piece of black vinyl tape, we are able to compare the SNRs of both systems. The Hilbert-transformed PA A-line profiles acquired by the OR-PAM I and II systems are shown in Figure 5c. The quantified SNRs measured by the OR-PAM I and II systems are 25 and 39 dB, respectively. Our results imply that the coaxial scanning approach of light and ultrasound using the OR-PAM II equipped with 2A-PDMS-WP-scanner (*i.e.*, called as 2A-PDMS-WP-OR-PAM) sufficiently improve the SNRs while maintaining the imaging speed, which is a key requirement for *in vivo* real-time PA imaging.

### 3.2. In Vitro Photoacoustic Imaging of Gold Micro-Patterns

**Figure 6:**
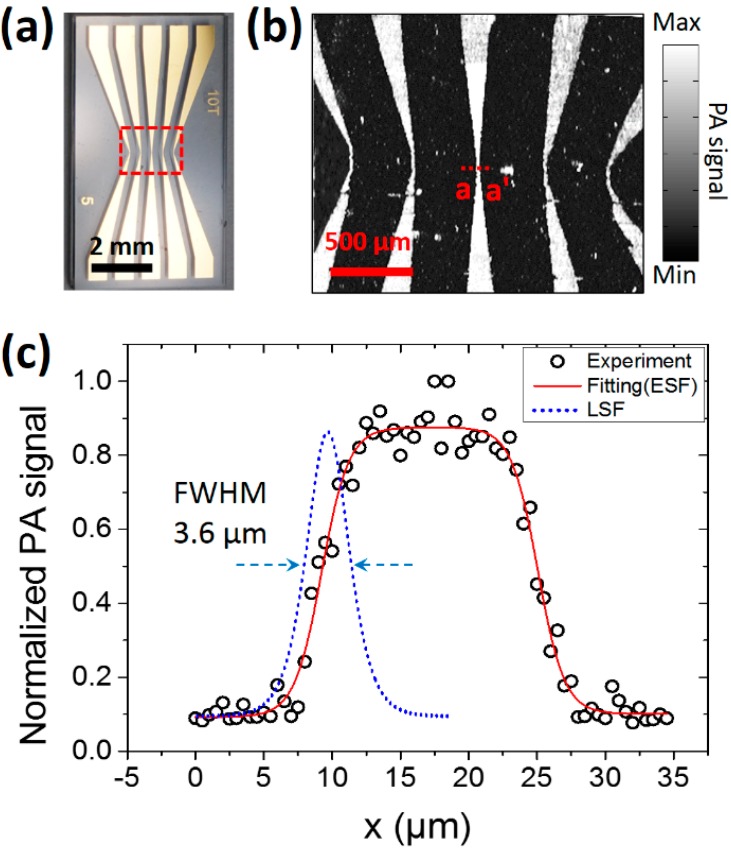
*In vitro* PA imaging of gold micro-patterns using the 2A-PDMS-WP-OR-PAM. (**a**) Photograph of gold micro-patterns; (**b**) PA maximum amplitude projection image of the red boxed region of (a); (**c**) PA amplitude profile across the a-a′ line of (b) and fitted edge spread function (ESF) and line spread function (LSF). Full width at half maximum (FWHM) in the plot means a lateral resolution of the system.

To verify the performance of the 2A-PDMS-WP-OR-PAM system, a gold micro-patterned sample with a thickness of 200 nm on a glass plate (Figure 6a) is photoacoustically imaged (Figure 6b) *in vitro*. The gold micro-patterns are prepared by a conventional MEMS process, which includes a photolithography for micro-patterning and etching the gold film on a glass substrate. The smallest width of the gold micro-patterns is 20 μm on the center. The scanning range is 2 mm × 1.8 mm along the X and Y axes, respectively. As shown in Figure 6b, the gold micro-patterns are clearly visualized in the PA image. To quantify the lateral resolution, the PA amplitude profile along the a-a′ line is plotted in Figure 6c. The line spread function (LSF) is derived from the fitting curve of the edge spread function (ESF) in the range of from 0 to 18 μm. The calculated full width at half maximum (FWHM) of the LSF, a lateral resolution of the system, is 3.6 μm, and the value well matches with the theoretical estimation (*i.e.*, 3.4 μm). The axial resolution is determined by the one-way acoustic bandwidth, approximately 26 μm [15]. 

### 3.3. In Vivo Photoacoustic Imaging of a Mouse Ear

**Figure 7:**
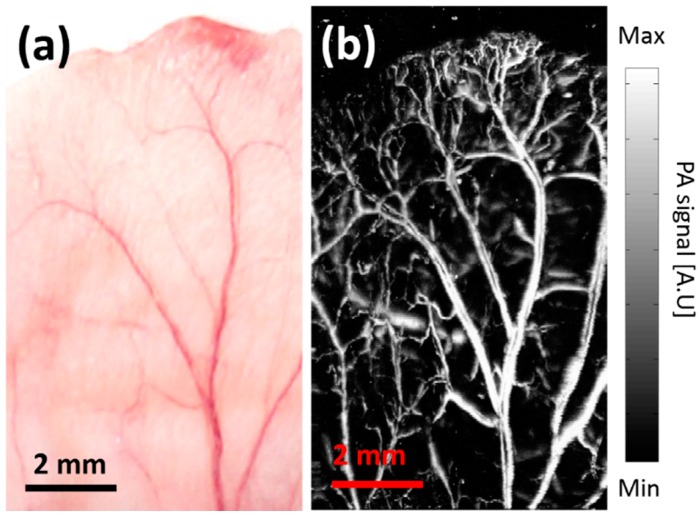
*In vivo* noninvasive PA imaging of the microvasculatures in a mouse ear. (**a**) Photograph of the mouse ear; (**b**) *In vivo* corresponding noninvasive PA image of the mouse ear.

Lastly, *in vivo* PA imaging of a mouse ear is conducted to verify the high SNRs, wide FOV, and fast scanning of the 2A-PDMS-WP-OR-PAM system. All animal experimental procedures satisfy with the laboratory animal protocol admitted by the institutional animal care and use committee of Pohang University of Science and Technology (POSTECH). A healthy Balb/c mouse (weighing ~20 g) is initially anesthetized and maintained with vaporized isoflurane gas. The fine hair on the mouse ear is removed by commercial depilatory, and then the mouse is positioned on a handmade animal holder. An electrical heating pad is used to keep the body temperature. The excited pulsed laser energy (*i.e.*, 13 mJ/cm^2^) is below the safety limit (*i.e.*, ~20 mJ/cm^2^) of laser usage on skins. To acquire dense pixels numbers (*i.e.*, 1000 pixels × 500 pixels along the X and Y axes, respectively), a triangular waveform with a frequency of 5 Hz and a voltage of 5 V is used for the B-scan image along the X axis. A sawtooth waveform with a frequency of 0.01 Hz and a voltage of 5 V is used for volumetric imaging along the Y axis. A volumetric PA image with the wide FOV (*i.e.*, 6.6 mm × 11 mm along the X and Y axes, respectively) is displayed within 100 seconds. Figure 7a shows the photograph of the mouse ear and the corresponding *in vivo* PA image is shown in Figure 7b. The PA image clearly visualizes large blood vessels as well as capillaries. The imaging speed of the 2A-PDMS-WP-OR-PAM system has been currently limited by the repetition rate of the used pulsed laser. We expect that volumetric PA imaging rate will be further enhanced by using faster pulse laser (*i.e.*, 500 kHz) and the optimized imaging processing. 

## 4. Conclusions

We have developed a 2-axis PDMS-based waterproof scanner and demonstrate its use in two forms of OR-PAM systems. The fabrication process of the scanner is detailed in each step. The main approach is soft lithography of PDMS, which has low stiffness and good water resistance. The fabricated scanner simultaneously and coaxially reflect both ultrasound and light in both air and water. The small size and simple operation using DC or AC driving voltages make the PDMS scanner versatile in different forms of OR-PAM systems. A gold micro-pattern is photoacousticlly imaged *in vitro* and finally, we successfully image microvasculatures of a mouse ear *in vivo* using the developed OR-PAM systems. This 2A-PDMS-WP-scanner can be potentially used for many types of OR-PAM systems such as handheld probes, endoscopic probes, laparoscopic probes, and so on. 
